# Mechanisms of Cognitive Impairment in Depression. May Probiotics Help?

**DOI:** 10.3389/fpsyt.2022.904426

**Published:** 2022-06-09

**Authors:** Maria Dobielska, Natalia Karina Bartosik, Kamil A. Zyzik, Edward Kowalczyk, Michał Seweryn Karbownik

**Affiliations:** ^1^Students’ Research Club, Department of Pharmacology and Toxicology, Medical University of Łódź, Łódź, Poland; ^2^Institute of Sociology, Jagiellonian University, Kraków, Poland; ^3^Department of Pharmacology and Toxicology, Medical University of Łódź, Łódź, Poland

**Keywords:** depression, cognitive impairment, mechanism, probiotics, gut-brain axis

## Abstract

Depression is the major cause of disability globally. Apart from lowered mood and accompanying symptoms, it leads to cognitive impairment that altogether predicts disadvantaged social functioning. Reduced cognitive function in depression appears a bit neglected in the field of clinical and molecular psychiatry, while it is estimated to occur in two-thirds of depressed patients and persist in at least one third of remitted patients. This problem, therefore, requires elucidation at the biomolecular and system levels and calls for improvement in therapeutic approach. In this review study, we address the above-mentioned issues by discussing putative mechanisms of cognitive decline in depression: (1) increased oxidative stress and (2) inflammation, (3) disturbed hypothalamus-pituitary-adrenals axis, and (4) reduced monoamines functionality. Moreover, we acknowledge additional underpinnings of cognitive impairment in depressed elderly: (5) vascular-originated brain ischemia and (6) amyloid-beta plaque accumulation. Additionally, by reviewing molecular, pre-clinical and clinical evidence, we propose gut microbiota-targeted strategies as potential adjuvant therapeutics. The study provides a consolidated source of knowledge regarding mechanisms of cognitive impairment in depression and may path the way toward improved treatment options.

## Introduction

Human cognition has been a subject of philosophical debate for millenia: from Plato’s search of a metaphysical truth behind superficial physical reality, through Descartes’ most well-known phrase of cogito ergo sum, up to contemporary instances of the mind-body problem. One may assert that the nature of our cognition is the very thing that makes us human. A loss in cognitive functions, frequent in depression, is therefore a vital issue for individuals and societies. Therefore, it is all the more important to understand the scale and nature of the problem.

Cognitive functions, which are mental processes of receiving, using and preserving information, may be classified into domains such as attention, perception or memory and also problem solving, reasoning, or planning (1). Impaired cognition is defined as more than one standard deviation below the mean score test result of the normative samples according to a test score to gender and age (2). Meta-analyses on symptomatology of depression have established that diverse cognitive functions were deteriorated in the course of depression, among others: alertness, psychomotor speed (3), attention, memory, executive functions (4) and verbal fluency (5). Cognitive deficits have been estimated to occur in around two-thirds of depressed patients (4); moreover, they persist among at least one third of remitted individuals (4). According to the World Health Organization, approximately 280 million people in the world suffer from depression (6). This makes cognitive impairment in depression a vitally important problem that requires elucidation at the biomolecular and system levels and calls for improvement in therapeutic approach.

Cognitive impairment in depression appears age-dependent (3). The recent meta-analyses suggested that young people have a greater chance for normalization of cognitive functions after resolution of depressive symptoms (3), whereas a vast majority of the elderly suffered from persistent cognitive deficits despite the remission of depression (7). Moreover, older age seems to be a risk factor for deeper cognitive impairment in depression (8). However, a more severe course of cognitive impairment was also associated with early onset of the disease (9).

Cognitive impairment is also associated with poor response to treatment (9–11) and is a significant relapse predictor (2). It poses questions about the influence of antidepressants on patients’ cognitive functions, which has not been clearly established (12). For instance, Lee et al. (13) demonstrated that among patients experiencing their first depressive episode antidepressants improved shifting (the cognitive process of adapting to change), however the medicines were also associated with worse results in memory and verbal learning tests (13). Tricyclic antidepressants were found to fail in improving cognitive functions, while vortioxetine and bupropion have demonstrated pro-cognitive effects in depressive patients as compared to selective serotonin- (SSRI) and serotonin-norepinephrine-reuptake inhibitors (14). Due to the fact that a vast majority of medication currently used to treat depression has either been associated with impaired cognition occurring after treatment, or have had a beneficial, but pseudo-specific, effect on cognition (15), further research on antidepressant-naive patients should be conducted (16).

Acetylcholinesterase inhibitors that are used primarily in Alzheimer’s dementia might raise some hope (17). However, a randomized control trial on effectiveness of donepezil in treatment of depression with coexisting cognitive impairment did not support using adjunctive off-label cholinesterase inhibitors (17). Depressive patients treated with donepezil not only did not achieve any improvement in cognition, but also experienced more adverse effects than the placebo group (17). It is also worth mentioning that using cognitive enhancers – also known as smart drugs – seems to be dangerous even for healthy individuals due to their uncertain compositions (18), risk of addiction and multiorgan side effects (19).

When it comes to non-pharmacological methods, their effectiveness is questionable. On the one hand, psychological, behavioral and somatic therapy seems to be promising in comparison to antidepressants in management of late-life depression, but on the other hand these findings might carry a risk of methodological limitations (20). Another study, which was not included in the above-mentioned review, undermined the credibility and raised doubts on the effectiveness of non-pharmacological methods – memory flexibility training performed in patients with major depressive disorder (MDD) did not lead to significant improvement in their cognition (21).

According to a comprehensive systematic review which considered assessment of cognitive impairment in depression and efficacy of different therapeutic methods, combinations of antidepressant monotherapy and novel antioxidant and anti-inflammatory adjuvants should be enabled to increase various cognitive domains. It is underlined that the field urgently requires more large-scale, randomized, placebo-controlled trials that include testing cognition as a primary outcome with adequate, ideally standardized, neurocognitive batteries (22). However, before performing clinical trials, the current knowledge on these adjuvants should be analyzed and summarized.

In our review, we analyzed the current research on mechanisms of cognitive impairment in depression in order to discuss a specific adjuvant therapeutic strategy: probiotics. Since problems in the gut-brain axis might be fundamental for either depressive, or cognitive symptoms, probiotics appear promising.

## Materials and Methods

Authors conducted a query in web databases in search of articles related to the subject of the study. The databases included mostly PubMed and Google Scholar. One hundred forty two articles were included in the study and analyzed.

## Mechanism of Impaired Cognition in Depression

A comprehensive analysis of the mechanisms in which cognition is impaired in the course of depression might help establish possible treatment options. The range of cognitive deficits widens with each consecutive episode of depression. Depression therefore seems to have a neurodegenerative component (15). This is the case particularly in the elderly, who are notable of white matter hyperintensities (23) and/or high amyloid-beta presence in the brain tissue (24). For them, cognitive deficits in depression might be a prodrome of Alzheimer’s disease (25), especially if associated with high amyloid-beta levels (26). On the other hand, Jamieson et al. (27) demonstrated that the mechanism according to which depression originates and develops often depends on a patient’s age. In the youth, the dominant psychosocial factors (28, 29), coexisting with a genetic predisposition (30), trigger a biological effect of chronic inflammation (31) and a decrease in hippocampal volume (32).

### Oxidative Stress, Inflammation and Impaired Neuroplasticity

Studies put emphasis on the inflammatory process that takes place in a depressive patient’s organism (33). Therefore, depression becomes increasingly often and boldly regarded as an inflammatory disease (34). Inflammatory processes are accompanied with and triggered by oxidative stress – a disproportion between free radicals production and antioxidant defense mechanisms (35). When the inflammatory response occurs within the central nervous system (CNS), it is called neuroinflammation (36). That can be caused by various pathological insults, including infection, trauma, ischemia and toxins. The process is marked by production of pro-inflammatory cytokines, tumor necrosis factor α (TNF-α), chemokines, small-molecule messengers, including prostaglandins and nitric oxide (NO), and reactive oxygen species by innate immune cells in the CNS (37).

**FIGURE 1:**
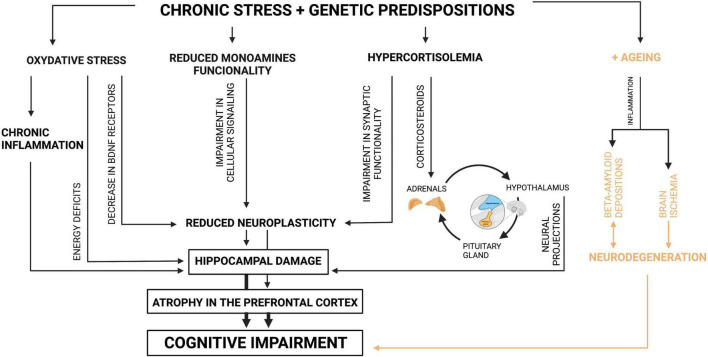
Mechanisms of cognitive impairment in depression. Source: authors; created in BioRender.com. Genetically predisposed individuals who are exposed to negative environmental factors, which induce depression, present a variety of changes in their organisms. A basic defensive reaction for environmental stress is oxidative stress, which results in energy deficits on the level of the cell. Particularly sensitive cells are the cells of the hippocampus; therefore its damage occurs as the consequence. Moreover, oxidative stress leads to decrease in the number of BDNF receptors, which manifests itself in reduced neuroplasticity. Oxidative stress also triggers inflammation, which as well results in damage in the hippocampus. Another reaction for chronic stress among genetically predisposed individuals is reduced monoamines functionality which by impairment in cellular signaling leads to reduced neuroplasticity. The next parallel reaction for chronic stress, possible because of specific genes, is hypercortisolemia. This one, when it is chronic, results in disturbance in the HPA axis. Due to the fact that the HPA axis functions in the feedback loop with the hippocampus, its disturbance leads to the hippocampus damage. Moreover, the increased level of glucocorticoids impairs synaptic functionality, which is manifested by reduction in neuroplasticity. In general, when neuroplasticity is affected, the hippocampus and prefrontal cortex present shrunk neurons, which leads to atrophy or even damage of the tissues. Due to the fact that these structures are mainly responsible for cognitive performance, their functional alterations result in cognitive impairment. Additionally, the elderly who are genetically predisposed and affected by environmental factors present brain ischemia derived from cardio-vascular problems and beta-amyloid depositions. Both pathomechanisms also manifest themself as cognitive impairment.

According to the literature, oxidative stress might be a primary reaction to disadvantageous environmental factors (34). In general, oxidative stress affects energy metabolism, which leads to a damage in DNA, RNA, lipids and proteins (37). A structure particularly sensitive to energy deficits is the hippocampus, especially its pyramidal neurons (Figure 1). The hippocampus is responsible for many cognitive functions; it is involved not only in memory formation and storage, but contributes to flexible cognition (38), which appears to play a role in recovery from depression (39). Therefore, oxidative stress is suggested to be responsible for cognitive impairment, particularly in the course of depression (40).

Moreover, oxidative stress causes a decrease in the expression of tropomyosin receptor kinase B (Trk B) reducing its function (41). This receptor is a target for brain derived neurotrophic factor (BDNF) – a protein involved in brain plasticity mechanism (41). Neuroplasticity is a widely used non-specific term, but the underlying theme is a change in the efficacy of connections between neurons. This change might occur in several mechanisms. One of the ways in which synaptic transmission could be altered is change in morphology of neurons (42). Research revealed that BDNF administration to cell cultures causes growth of serotonergic neurons and dendrite lengths (43). Therefore, oxidative stress, typical for depressive patients, may affect neuroplasticity, resulting in neuronal atrophy and synaptic loss in the medial prefrontal cortex and the hippocampus (44) (Figure 1).

What is more, oxidative stress triggers an inflammatory response (41) (Figure 1). Initially, basic immune factors such as C-reactive protein (CRP), interleukin 1 (IL-1) and interleukin 6 (IL-6) were found to be elevated in depression (45). Moreover, some more in-depth studies revealed an increase in the concentration of chemokine such as C-C motif chemokine ligand 2 (CCL-2) among patients with depression. The CCL-2, an immune modulator with numerous receptors in the CNS, is recognized to have a neuromodulatory effect. Not surprisingly, the same immune factor was proven to be elevated among patients suffering from mild cognitive impairment (MCI) (46). CCL-2 might also be involved in the altered metabolism of beta-amyloid underlying Alzheimer’s disease (AD). Its elevated level was associated with cognitive impairment as noted by worse results in the Mini Mental State Examination (47). This favors the view that inflammation has an influence on the pathophysiology or even the pathogenesis of cognitive impairment in depression.

In turn, microglia is responsible for maintaining and modulating connectivity of the brain, but microglial overstimulation is closely associated with synapse elimination (41). For that reason, an excessive stimulation of microglia might cause an imbalance in the release of pro-inflammatory cytokines stimuli and neurotoxic products (41). The pro-inflammatory phenotype releases TNF-α, interleukin 1β and IL-6, which are neurotoxic signals and cause neurodegeneration in the hippocampus by contributing to neuroinflammation (41). Interestingly, *Bacteroides fragilis* and *Escherichia coli* secrete lipopolysaccharide (LPS), which was found as causing an increase in the level of proinflammatory cytokines (48). That might be harmful to neuronal integrity, which is normally maintained by astrocytes (48). In the study performed by Garcez et al. (48) it was revealed that sodium butyrate and indole-3-propionic acid, which are also bacterial-derived metabolites, and they can manage to lower the proinflammatory cytokines levels which was produced by LPS-activated astrocytes (48).

### Dysregulated Hypothalamic-Pituitary-Adrenal Axis and Its Effect on the Hippocampus

Another mechanism is based on a dysfunction in the hypothalamic-pituitary-adrenal (HPA) axis. Research found that a disturbance might be secondary to gene polymorphism (49), exposure to maternal depression in childhood (50) or experience of early life stress (51) (Figure 1). In general, studies share a view that depressive patients presented hypercortisolemia (52–54), unless they had an atypical depression (55). Elevated cortisol levels were revealed to cause at least partial impairment in autobiographical (56) or long-term declarative memory (57), whereas acute anti-rheumatoid therapy with glucocorticosteroids may induce memory retrieval impairment (58).

Due to the fact that glucocorticoids are information carriers in the feedback loop between adrenals and hippocampus, hypercortisolemia was hypothesized to cause hippocampal damage (54). More in depth studies revealed that chronic psychological stress and consequential hypercortisolemia suppress neurogenesis in hippocampal cells and lead to hippocampal atrophy (59, 60). This process is mediated by corticosteroids, which have their receptors in the hippocampus (59, 60) (Figure 1).

On the other hand, according to a less popular hypothesis, the above causality could be of inverse direction (52). Researchers considered if the hippocampal abnormalities itself might trigger hypercortisolemia in depression (52). Finally, the hippocampus provides an important source of negative modulation of the HPA stress hormone axis through its projections to the hypothalamus (60). Hippocampal dysfunction therefore may contribute to the dysregulation of the stress response that is seen in depression (60).

Despite the fact that a consensus has not been established yet, both the HPA-axis and hippocampus are responsible for proper cognitive functioning and both are affected in depression.

### Coexistence of Excessive Oxidative Stress and Hypothalamic-Pituitary-Adrenal-Axis Dysregulation

**FIGURE 2:**
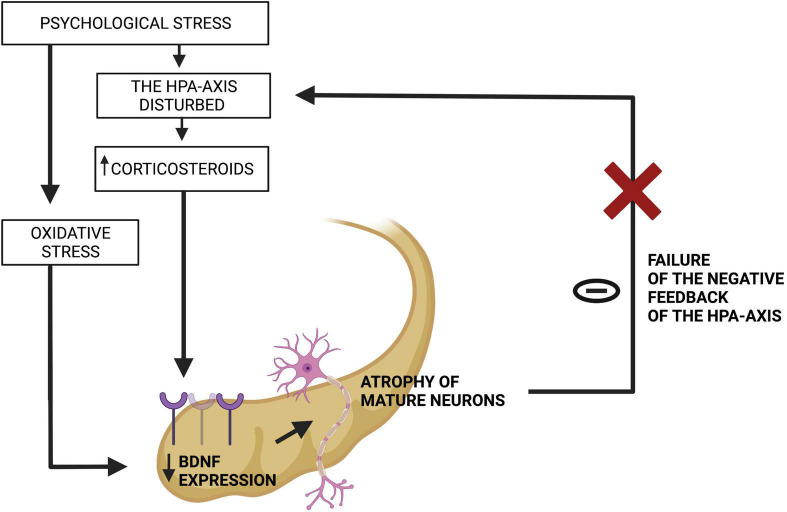
Failure of the negative feedback loop between the hippocampus and the HPA-axis. Source: authors; created in BioRender.com. In genetically predisposed individuals, a hostile environment leads to psychological stress, which in its chronic form impairs the HPA-axis and triggers oxidative stress. They both decline BDNF expression in the hippocampus, which atrophies this organ and makes it unable to fulfill its function of suppressing the HPA-axis.

It is also worth mentioning that the hippocampus provides negative feedback of the HPA axis (59) (Figure 2). Therefore, when the hippocampus is atrophied, the HPA axis is disturbed and the level of glucocorticoids grows (59). Excessive amount of glucocorticoids, as well as the oxidative stress, leads to decrease in BDNF expression (59). When BDNF level is insufficient, the hippocampus undergoes further atrophy, which results in failure of negative feedback of the HPA-axis (59). This leads to further atrophy of the hippocampus, which closes the vicious circle (59). Additionally, it indicates a connection of oxidative stress and disturbed HPA axis in the pathomechanism of cognitive impairment in depression (59).

### Morphological Abnormalities

Decrease in hippocampal volume appears to cause memory dysfunction (21, 54). This claim sounds coherent, as the hippocampus has been established to provide cognitive functions such as working and episodic memory (61, 62). Interestingly, impaired activity in hippocampal subfields has been associated with subclinical depressive symptoms, while greater structural damage in the hippocampus has been associated with greater severity of depression (27). Changes in the hippocampus have been suggested to be caused also by inflammatory processes (32, 63). This sustains the causal pathways between the above hypotheses.

Generally, a chronic stress underlying depression leads to a variety of changes in morphology, also in other structures than the hippocampus (60). For example, the prefrontal cortex undergoes atrophy, whereas amygdala is hypertrophied (60). The first change leads to attention and behavioral task difficulties, second impairs fear-dependent learning (60). Also some reduction of brain tissue in the thalamus and basal ganglia was noticed among depressive patients (43). More in depth studies established that stress activates the HPA axis and leads to neuroinflammation (43). Neuroinflammation results in an hyperactivation of NMDA-receptors, then – as a consequence – a neuronal damage, and eventually an impairment in synaptic functionality (43).

### Impaired Monoamines Functionality

Synaptic plasticity might be also affected by reduced functionality of monoamines, typical for depressive patients (60) (Figure 1). It might be explained by the fact that physiologically, serotonin, noradrenaline and dopamine by binding to their receptors trigger the cAMP production and then stimulate production of protein kinase A and finally transcription factors in neurons (60). Those transcription factors are responsible for expression of genes which promote neurogenesis (60). Therefore, when functionality of monoamines is reduced, cellular signaling is impaired and so neuroplasticity is (60). It is in line with another study on depression, which proposed that abnormalities in monoamines impaired cellular signaling and neurocircuit pathways, which led to cognitive impairment (64).

### Amyloid-Beta Deposits

There is still no consensus about the involvement of amyloid-beta in the development of late-onset depression (27). So far, amyloid-beta has been shown to be responsible for cognitive impairment (65). Indeed, research revealed that among patients older than 65 years, those who were diagnosed with depression had the amyloid-beta levels significantly higher in comparison to the non-depressive group (65). Moreover, these depressive patients, who had increased amyloid-beta levels in plasma, suffered from a greater degree of longitudinal cognitive decline than amyloid-beta-negative patients (65). A preclinical study revealed that artificially induced neuro-inflammation contributes to the accumulation of beta-amyloid in the hippocampus and cerebral cortex of mice brains and to the consequential memory impairment (66) (Figure 1). Due to the fact that neuro-inflammation seems to underlie both amyloid-beta deposition and depression, AD and depression are increasingly often described as closely associated diseases (67). The key role has transforming growth factor β1 (TGF-β1) – an anti-inflammatory cytokine that exerts neuroprotective effects against amyloid-beta induced neurodegeneration (67). TGF-β1 plasma levels are reduced in major depressed patients and correlate with depression severity and significantly contribute to treatment resistance (67). The deficit of TGF-β1 signaling is also responsible for synaptic dysfunction and memory loss and is an early event in AD pathogenesis (67).

### Brain Ischemia

The elderly often experience problems regarding the cardio-vascular system, which are mainly derived from arteriosclerosis (27). An escalating ischemia causes white matter hyperintensities in magnetic resonance imaging (MRI) that contribute to impairment in multiple cognitive domains, such as global cognition, episodic and working memory, executive functions and processing speed (27) (Figure 1). In another study performed on the elderly, both depressive and non-depressive participants presented similar levels of cerebrovascular risk (68). Probably because of that, white matter damage, visualized in MRI, occurred in both groups of patients (68). This result was somewhat unexpected by the researchers, because higher white matter hyperintensities was reported as a feature of late-life depression in many previous reports (68). However, in patients with clinical depression the burden of white matter hyperintensities was associated with functional changes during affective processing (68).

As it has been discussed above, our knowledge about precise mechanisms contributing to cognitive impairment in depression remains inconclusive. However, they seem to be firmly interconnected on various levels of causation (Figure 1). We presented those mechanisms separately, because their complexity limited the explanatory potential of a collective description. We are aware that the proposed fragmentarization restricts the view on the whole problem and might bring about an impression that the mechanisms are independent from one another. However, we believe that the separate description of each mechanism allows for a thorough analysis. Nevertheless, it is essential to focus also on how they are interconnected. Many of these mechanisms can be influenced by gut microbiota-targeted interventions. This will be discussed in next paragraphs.

## Probiotics as a Potential Therapy for Cognitive Impairment in Depression

Mechanisms which underlie cognitive impairment in depression are complex and interconnected, therefore an effective treatment should be adjusted to this. Due to the influence of the gut on the brain, a promising therapy for neuropsychiatric diseases might include microbiota-targeted interventions. The brain-gut axis employs the CNS, neuroendocrine and neuroimmune systems, the autonomic nervous system, the enteric nervous system and the gut microbiota (69). This axis connects pathways in the immune, endocrine and neural systems (70). Therefore, a change in the gut microbiota brings changes in a variety of brain structures.

Homeostasis of brain functioning might be rebalanced by microbiota-targeted interventions, which include probiotics, prebiotics and synbiotics (71). According to a current definition, probiotics are living microorganisms that, when administered in adequate amounts, confer a health benefit on the host (72). Probiotics have an impact on the host’s intestinal microbiome (73), which is defined as the collective genetic information contained within the microbiota, including bacteria, viruses and fungi, residing in and on the human body (74). Moreover, they are selectively used by the host’s microorganisms, while not being harmed by the host’s enzymes (69). In turn, prebiotics are defined as a non-digestible food ingredient that benefits the host by selectively stimulating growth or activity of particular bacteria in the colon (75). Synbiotics are probiotic supplements containing prebiotic components (76). Discoveries regarding the influence of human microbiota on brain functioning led to the creation of a category of psychobiotics – a term coined by Dinan, Stanton, and Cryan (77); it includes probiotics and prebiotics that have a positive impact on mental health upon ingestion in appropriate amounts (69). Psychobiotics can improve mood and cognition and reduce anxiety and stress (38) through modulation of the microbiota-gut-brain axis signaling (38, 48).

### Oxidative Stress and Inflammation

One of the mechanisms which might impair cognition among depressive patients is excessive oxidative stress. It triggers an inflammatory reaction, which contributes to the damage of brain structures, such as the hippocampus (41). Therefore, presumably, a reduction of oxidative stress should alleviate the progression of cognitive decline.

#### Mechanisms of Antioxidant Properties of Probiotics

**FIGURE 3:**
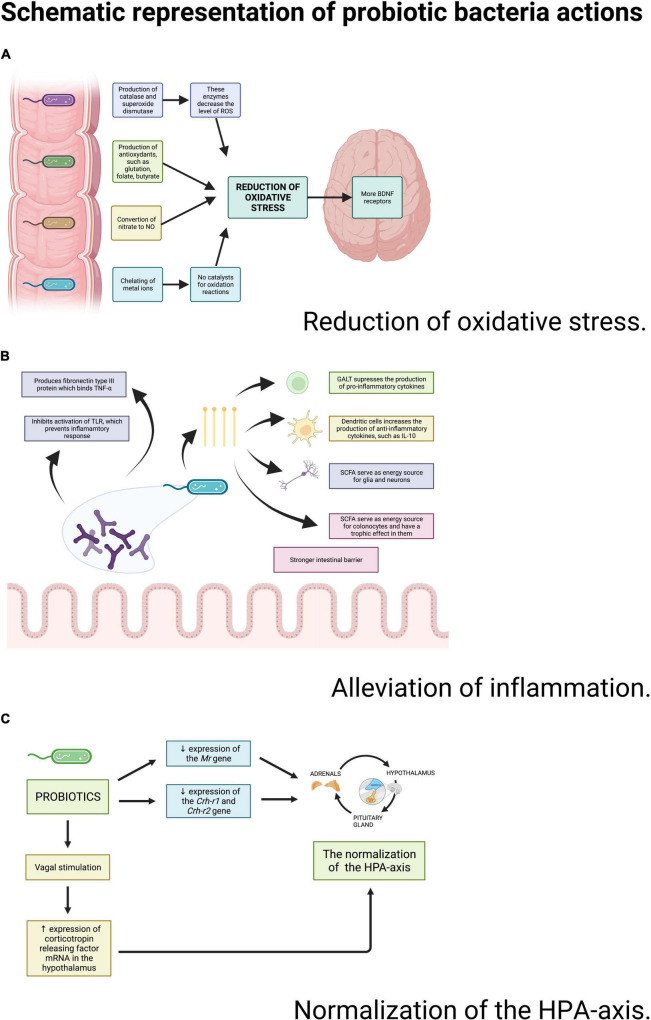
Schematic representation of probiotic bacteria actions. Source: authors; created in BioRender.com. Probiotic bacteria may reduce the oxidative stress, decrease the inflammation and improve the functioning of the HPA-axis through a wide variety of actions.

Antioxidant properties of probiotics are well-documented and multi-dimensional (76) (Figure 3). Probiotics suppress oxidative stress by producing glutathione (78), folate and butyrate, which are metabolites with an antioxidant potential (76). *Lacticaseibacillus rhamnosus* GG suppresses the immoderate proliferation of some bacteria by a certain low-molecular-weight substance secretion. In that mechanism, it might bring about a reduction in oxidative stress (76). Some bacteria strains produce catalase and superoxide dismutase enzymes. Both of these enzymes decrease the levels of reactive oxygen species (ROS), which makes probiotics antioxidants (76). *Lactobacilli* can also convert nitrate to NO, which is suggested to suppress oxidative stress by inhibiting mitochondrial respiration (40). Additionally, probiotic bacteria are involved in many signaling pathways with antioxidant properties such as: NFκB (78), Nrf2-Keap1-ARE, MAPK, and PKC (76). It was observed that some *Lactocaseibacillus* species can regulate cyclooxygenase-2 expression in thymus macrophages and reduce NADPH oxidases (NOX) activity, which are both responsible for generating ROS (76). Finally, probiotics have an antioxidant ability of chelating metal ions, especially Fe2+ and Cu2+ to prevent them from catalyzing the oxidation reaction (76).

#### Mechanisms of Anti-inflammatory Properties of Probiotics

Anti-inflammatory properties of probiotics have also been broadly discussed (Figure 3). The gut microbiota impacts the immune system and straightens the intestinal barrier. Probiotics produce short-chain fatty acids (e.g., butyrate) (79) that activate G protein-coupled receptors responsible for suppressing the production of pro-inflammatory cytokines (41). Probiotics do not change the general amount of cytokines, but they shift their proportions: the level of proinflammatory IL-6 and TNF-α decreases in relation to the increasing level of IL-2, IL-4, and IFN-γ (80). Moreover, *Bifidobacteria* produce fibronectin type III domain (FN3) protein, which is capable of binding TNF-α (81). These species are also capable of preventing the inflammatory response by inhibiting the activation of Toll-like-receptors (TLR) (69).

#### *In vitro* and Preclinical Trials Considering Probiotics and Prebiotics as Agents That Might Improve Cognition Due to Their Antioxidant and Anti-inflammatory Properties

Oxidative stress decreased the number of receptors for BDNF, a protein involved in neuroplasticity (41). Studies on animals proved probiotics administration to be associated with better cognitive performance and increased expression of BDNF (82, 83). Similar results were obtained by using prebiotics – fructans obtained from agave, called agavins (35). Mice fed with agavins had lower oxidative stress (evaluated by level of lipid peroxidation and carbonyls) detected in the hippocampus and cerebellum (35). These mice also had increased BDNF levels in the cerebellum (35). A review also indicated that probiotics facilitate the normalization of BDNF expression levels and normalize anxiety-like behavior (84).

Probiotics, such as *Limosilactobacillus reuteri, Lacticaseibacillus rhamnosus*, and *Bifidobacterium infantis*, decreased oxidative stress in rats which had the amyloid-beta injected into the hippocampus (85). It was triggered by reducing malondialdehyde and increasing levels of antioxidant enzymes, such as superoxide dismutase (85). Neuro-inflammation also abated, which manifested itself in a decrease in IL-1β and TNF-α in hippocampal tissue (85). Moreover, both groups of rats (injected and not-injected with beta-amyloid), when nourished by probiotics, improved their spatial memory (85). In another study on rats, prolonged administration of probiotics also weakened cytokine response to mitogen stimulation and reduced apoptosis susceptibility in the limbic system (86).

Research revealed that long-term probiotics administration to senescent mice changed their behavior into less preservative and improved their memory, which – among others – manifested in further walks (83). The behavioral changes might be once again explained by a detected increase in antioxidative enzymes and anti-inflammatory cytokines and a decrease in inflammatory markers (83).

The last two trials (83, 85) made an attempt to achieve conditions similar to these in which cognition is impaired also in humans. They stimulated amyloid-beta presence in the brain to mimic AD (or late-onset depression) and used old animals to account for aging (83, 85). These studies revealed that microbiota-targeted interventions improve cognitive functioning in the animals (83, 85). Therefore, it brings hope about therapy for humans.

However, it is also worth mentioning that the aging process might influence the (generally beneficial) role of microbiota in terms of oxidative stress. The study on young-adult and aged mice proved that those old mice which had been housed under germ-free conditions showed a diminished oxidative stress and ameliorated mitochondrial dysfunction in microglia in comparison to the mice with retained microbiota (87). The association was assumed to be explained by a toxic metabolite derived from gut microbiota - N6-carboxymethyl lysine, which were found in the microglia of aging mice, but not in young-adult mice (87). Nevertheless, those mice were not supplemented with probiotics, which are supposed to be beneficial by definition or at least not toxic, but housed in laboratory conditions, therefore the quality of their gut microbiota could be disturbed (87).

### Dysregulated Hypothalamus-Pituitary-Adrenal Axis

Another mechanism in which a hippocampal damage occurs is hypercortisolemia triggered by a dysfunction in the HPA axis. Research indicated that administration of probiotics could alleviate the abnormalities.

#### Mechanisms in Which Probiotics Influence the Hypothalamic-Pituitary-Adrenal Axis

Initially, it was revealed that adrenocorticotropin and corticosterone were more elevated in the group of germ-free mice than in the house-specific pathogen-free individuals (84). This confirms the theory that the HPA reactivity and the gut microbiota are codependent (84). More in depth studies revealed the causes of this dependence. In a study conducted on rats, it was observed that probiotics reduced expression of the Mineralocorticoid receptor (Mr), Corticotropin-releasing hormone receptor 1 (Crh-r1), Corticotropin-releasing hormone receptor 2 (Crh-r2) genes, transcripts of which are involved in regulation of the HPA axis (80). Moreover, the intestinal microbiota can activate vagal pathways that have their afferent endings in the gut (84). In rodents, a vagal stimulation caused an elevation of the expression of corticotropin releasing factor mRNA in the hypothalamus, resulting in a normalization of HPA-axis parameters (69) (Figure 3).

#### *In vitro* and Preclinical Trials Considering Probiotics and Prebiotics as a Therapy for Excessive Stress Response

Systematic reviews showed that probiotics can impact the HPA axis by lowering the corticosterone and adrenocorticotropic hormone blood levels (81, 88). For instance, *Bifidobacterium infantis* tames the overactivity of the HPA axis (89). In another study, it was found that urinary free cortisol level decreased in rats after a month-long supplementation of probiotics and daily administration of probiotics significantly reduced anxiety-like behavior of the animals (90). Other researchers performed a surgery on rats to achieve a reversible myocardial infarction and a consequential decline in rat’s mood-related signs (91). Their findings indicated that a high-n-3 polyunsaturated fatty acids (PUFA) diet or an addition of probiotics to a low-n-3 PUFA diet was helpful in reducing apoptosis in the limbic system after a very stressful event and in attenuating depression-like behavior (91). It was worth mentioning that PUFA has been recently described as having prebiotics properties (92). Therefore, a hypothesis that consumption of probiotics and prebiotics might inhibit progression of cognitive symptoms in depression related to hypercortisolemia seems to be mechanistically plausible.

However, a group of scientists found no difference in the expression of corticotropin-releasing factor in the hypothalamus between the control group of rats and the animals, which had been exposed to stress by separation from their mothers (86). Despite this, they achieved an alleviation in depression-like behavior and a normalization of immune response following probiotic supplementation (86).

Therefore, it appears the microbiota-targeted interventions bring improvement in the mood-relevant indices in animals. These changes were generally associated with the normalization of the HPA-axis. However, in one study (86), there was no difference in the HPA-axis parameters, but the level of inflammatory cytokines was decreased instead (86). This leads to a conclusion that the benefits from the microbiota-targeted interventions might derive from both the immune or endocrine systems. Possibly, depending on the case, one system might be more prone to changes than another. It reminds us about the interconnections between those systems in the functioning of the brain-gut axis.

### Impaired Monoamines Functionality

Depressive symptoms have been broadly accepted to result from a decrease in levels and functions of monoamines (59).

#### Mechanisms in Which Probiotics Influence Monoamines

Probiotics have a broad impact on metabolism and functioning of monoamines (88). Prolonged supplementation of probiotics increases the peripheral tryptophan concentration as revealed by altering the levels of 5-hydroxyindoleacetic acid (5-HIAA) and dihydroxyphenylacetic acid (DOPAC) in the brain (88). Probiotics also influence the biochemistry of the CNS by changing c-Fos, dopamine and 5-HT levels (88). After probiotics intervention, c-Fos mRNA expression in the hippocampus was increased, while the level of dopamine and 5-HT grew (the vagus nerve has been proposed as a pathway for probiotics effects) (88). Moreover, the hippocampal expression of mRNA receptors, 5HT1A and 5HT2C, is also increased by probiotics (84). Additionally, intestinal microbiota are also capable of producing GABA, which – as an inhibitory neurotransmitter – decreases activity in the nervous system (89). The alterations described above suggest that probiotics contribute to an improvement in monoamines functionality by increasing their levels or numbers of receptors.

However, one study argued that probiotics accelerated the catabolism of tryptophan into the kynurenic and quinolinic acids (93). This reaction is dependent on the indol-amine-2,3-dioxygenase enzyme, which activity is elevated by lactobacilli (93).

#### *In vitro* and Preclinical Trials Considering Probiotics as a Therapy for Reduced Monoamines Functionality

According to research, a 2-week-long administration of *Bifidobacterium infantis* increased the levels of plasma tryptophan (69). A rise in the production of tryptophan (a substrate for serotonin production) contributes to a rise in serotonin levels (94). Due to the fact that probiotics increase the levels of monoamines, such as serotonin or dopamine, their long-term administration might be threatened by serotonin syndrome or psychotic symptoms. However, scientists who searched for these adverse effects did not notice any abnormalities on mice models (91).

Interestingly, in the hippocampi and striata of germ-free mice, an increased level of serotonin was observed (84). Further research is needed to acquire more detailed knowledge regarding the changes in serotonergic signaling (84) as serotonergic neurotransmission and level may have more complex implications with mood (95). For example, according to the clinical trial considering SSRI therapy, in all patients with MDD a significant decrease in 5-HT levels over treatment was noticed (95). The decrease was significantly more prominent in patients who responded to therapy compared to non-responders. Therefore, a successful SSRI treatment is associated with greater decreases in circulating 5-HT (95).

Moreover, the delivery of oligosaccharides which are prebiotics present in human or bovine milk to pigs was associated with an improvement in their recognition memory (96). Additionally, pigs fed with human milk demonstrated elevated volumes of left and right cortex (96). Researchers tried to explain this phenomenon by changing the expression of GABAergic and glutamatergic genes, which were frequently associated with the delivery of the oligosaccharides from human milk (96). These genes improve long-term potentiation in pathways responsible for memory (96). Furthermore, it was found that vagotomy prevented the changes described above, so the vagus nerve seems to mediate in the improvement in memory (96).

### Amyloid-Beta Deposits

It has also been hypothesized that cognitive impairment in depression, especially late-onset depression, might be related to the presence of amyloid-beta in the brain (27).

#### Mechanisms in Which Probiotics Might Suppress Amyloid-Beta Deposition

Apart from the genetic predispositions, also glial inflammatory reactions are contributors to amyloid-beta aggregation in the brain, which occurs among patients with depression and AD (67). Therefore, a suppression of oxidant stress and inflammation achieved through microbiota-targeted interventions might result in an improvement.

#### *In vitro* and Preclinical Trials Considering Probiotics as a Therapy for Amyloid-Beta Deposition

Mehrabadi and Sadr in the study mentioned above revealed that probiotics inhibit amyloid-beta deposition in the hippocampi of rats which had amyloid-beta injected into their hippocampi (85). Probiotics were more effective in the prevention of amyloid-beta plaque deposition than rivastigmine – a medicine typically used in AD (85). An analogical study on rats also revealed an inflammation reduction, a lesser number of amyloid-beta plaques and a better spatial memory in groups treated with probiotics (97).

### Brain Ischemia

#### Mechanisms in Which Probiotics Might Alleviate Brain Ischemia

When it comes to cognitive impairment in depression which might develop as a result of brain ischemia, probiotics have a more indirect potential of being useful (98). Probiotics are thought to enhance metabolism of lipids, suppress development of arterioslerotic plaques and reduce absorbtion of cholesterol (98). Thus, they are hypothesized to be an effective therapy option against arteriosclerosis (98). Therefore, if probiotics may prevent arteriosclerosis-related ischemia, they might as well prevent ischemic-related cognitive impairment.

## Clinical Studies Considering Probiotics as a Therapy for Cognitive Impairment

### Depression

Thorough analyses of many studies revealed that only one research team assessed cognitive functions of depressed patients in relation to gut microbiota-based interventions. Rudzki et al. (99) performed a randomized and double-blind, placebo-controlled trial on 79 patients with major depressive disorder (MDD) during their SSRI therapy (99). Research revealed that patients who underwent an additional 8-week long supplementation of probiotic bacteria *Lactobacillus plantarum* 299v improved their cognitive performance in the domains of attention, perceptivity and verbal learning in comparison to the placebo group (99). Looking for an explanation, they measured patients’ plasma levels of tryptophan, kynurenine and its metabolites, inflammatory cytokines and cortisol (99). The only significant change found was a decrease of kynurenine level, which could have contributed to the improvement of cognitive functions (99). Kynurenine develops from tryptophan in a parallel pathway to serotonin biosynthesis (99). Change from tryptophan to kynurenine occurs when specific enzymes are activated. Activation of these enzymes is triggered by pro-inflammatory cytokines, bacterial lipopolysaccharides, glucocorticoids or oxidative and nitrosative stress (99). The findings support the hypothesis discussed throughout the present study, as we also specified these factors as causes of cognitive impairment in depression and potential targets for probiotic therapy. To our knowledge, the results of this study are the first recorded evidence of improvement in cognitive functions of depressive patients achieved with probiotic bacteria supplementation (99).

Another double-blind controlled trial on 39 individuals with MDD was performed (100). The research revealed that patients supplemented with probiotics had decreased levels of C-reactive protein and an increase in antioxidant glutathione in comparison to a control group (100). These patients experienced a significant relief in depressive symptoms, assessed using the Beck’s Depression Inventory (100). Although cognitive functions were not directly tested, the tool included cognition-related questions regarding issues such as effectiveness of work or decision-making abilities (101).

A meta-analysis on twelve studies regarding various psychiatric diseases – from depression and AD to schizophrenia – also revealed that supplementation of probiotics decreased levels of CRP and malondialdehyde among neuropsychiatric patients (102). The level of anti-inflammatory IL-10 was found to be increased (102). Combining four studies on depression, a significant reduction in the Hamilton Depression Rating Scale following the probiotic supplementation was found (102). Again, cognitive functions were not directly tested (102). Nevertheless, the tool assessed cognitive-related matters such as retardation (slowness of thought and speech, impaired ability to concentrate and decreased motor activity) and ability to work and perform daily activities (103).

A meta-analysis on the use of probiotics to alleviate depressive symptoms (94) shows no result in healthy individuals, in contrast to the group with mild to moderate manifestation of depression, where probiotics alleviate the symptoms. It also revealed that administration of probiotics appeared to have no side effects (94). They also had no addictive properties (73).

In the matter of the elderly, a meta-analysis showed that patients older than 65 experienced no relief in depressive symptoms after implementation of probiotics, although younger participants achieved reductions in depressive rating scales (89). It might be explained by the age-dependent differences in pathophysiology of depression and cognitive impairment in depression (3) (Table 1). Among the young depressive patients inflammation is a dominant problem. In turn, the elderly additionally presents brain ischemia and beta-amyloid depositions, which might be consequences of chronic inflammation. Ongoing inflammation better responds to probiotics therapy than its consequences, therefore the probiotics therapy might be more effective in the young patients. Among the elderly depression has features of a neurodegenerative and thus irreversible disease.

**TABLE 1:** Age-dependent mechanisms of cognitive impairment in depression.

Pathomechanism	The young	The elderly
Genetic background	+	+
Psychosocial factors	+	
Excessive oxidative stress	+	+
Inflammation	+	+
Disturbed HPA axis	+	+
Monoamines abnormalities	+	+
Beta-amyloid depositions		+
Brain ischemia		+

*“+” indicates the presence of a particular pathomechanism.*

However, it is worth mentioning that in the aforementioned meta-analysis (89) only one study was performed on patients older than 65 (104). The diagnosis of depression was not an inclusion criterion to the study, nor was a diagnosis of any other psychiatric disease (104). Depressive symptoms were detected before and after a 20-week-long probiotics supplementation and there were no significant changes: neither in mood, nor in fatigue and confusion (104). It corresponds with a meta-analysis on effects of probiotic administration in depression which revealed improvements in depressive symptoms only in clinically depressed cohorts (105).

### Anxiety

Anxiety and depression are also closely associated and commonly coexisting diseases (106). Moreover, anxiety often progresses into depression (106), while high levels of anxiety are associated with poor memory (107).

An interesting study was performed simultaneously on animals and humans (90). Daily subchronic administration of probiotic formulation significantly reduced anxiety-like behavior in rats and alleviated psychological distress in human volunteers (90).

However, it doesn’t correspond with the results of a 30-day administration of *Saccharomyces boulardii* CNCM I-1079 in medical students in a stressful period of end-of-term examination session (108). That intervention revealed no significant difference in students’ cognitive performance under stress between students supplemented with probiotics and a placebo group (108). In another study, use of probiotics correlates with decreased values in the Depression Anxiety Stress Scale (DASS) in patients with anxiety, however, in other inventories measuring anxiety, such as State-Trait Anxiety Inventory (STAI) and the Hospital Anxiety and Depression Scale (HADS), there was insufficient evidence of anti-anxiety effects of probiotics (109). According to a meta-analysis regarding young people aged from 10 to 24, there is limited evidence for using probiotics as a treatment in this group, because the results concerning the reduction of stress by probiotic intervention were mixed (110).

### Alzheimer’s Disease

Antioxidant properties of probiotics allow them to be used in diseases characterized by excessive oxidative stress and neuro-inflammation. One of these diseases is AD, which manifests itself as a cognition impairment and is caused by beta-amyloid plaques in the CNS (67). That makes it similar to cognition impairment in depression (67). In the last several years, a large number of studies have demonstrated the neurobiological and clinical continuum between depression and AD (67). It was revealed that depressive patients present a decreased level of anti-inflammatory cytokine TGF-β1, that exerts neuroprotective effects against beta-amyloid-induced neurodegeneration (67). Therefore, the inference from characteristics of AD about cognitive impairment in depression seems reasonable.

A few studies were performed on humans diagnosed with AD (111–113) or MCI (114, 115). A meta-analysis concerning these studies revealed that administration of probiotics predicted the reductions in malondialdehyde and C-reactive protein (116). A significant improvement in cognition was noticed among patients who used probiotics in comparison to a control group (116). Patients treated with probiotics showed enhanced performance in the following tests: Mini Mental State Examination (MMSE), Verbal Learning Test, Auditory Continuous Performance Test and Digit Span Test (116).

For example, in a 12-week controlled clinical trial that recruited patients diagnosed with AD, a test group received 200 ml probiotic milk per day which contained *Lacticaseibacillus acidophilus, Lacticaseibacillus casei, Bifidobacterium bifidum*, and *Limosilactobacillus fermentum* (112). It resulted in an improvement in MMSE score in comparison to a control group that received regular milk (112).

In another study, a kefir containing various species of bacteria and fungi was used (117). After its 3-month-long supplementation, an improvement in attentive, executive and language functions, visual-spatial function, abstraction and constructive abilities and memory was noticed in elderly patients with AD (117).

However, besides the fact that a suppression of oxidant stress and inflammation achieved through microbiota-targeted interventions resulted in an improvement in the aforementioned studies, this kind of therapy is considered as an alternative (118). The efficacy of anti-inflammatory therapies remains unclear so far (118).

### Mild Cognitive Impairment

Cognitive impairment has a similar origin as various neuropsychiatric diseases (9). Indeed, it is considered a prodrome of AD (25). Patients diagnosed with MCI, as well as depressive patients, present a decreased hippocampal volume (9, 59). Hwang et al. (114) revealed that patients with MCI achieved an increase in serum BDNF after a 12-week-long supplementation of probiotics (114). These patients also experienced a significant improvement in combined cognitive functions, especially in the domain of attention (114). Cognitive improvement was associated with increased BDNF levels after consumption of probiotics (114).

What is more, a higher prevalence of *Bacteroides* was noted in the gut of MCI individuals than in those without dementia, but lower than in AD patients (119). The effects of probiotic administration in the elderly included changes in the composition of intestinal microbiota, especially by promoting growth of *Bifidobacteria* and *Lactobacilli* (119). Probiotics improved cognitive performance in AD and MCI patients (119). However, in a meta-analysis consisting of the same data, but concerning only individuals with AD, there was no beneficial effect observed (120). Nowadays, it is unknown how long the beneficial effect lasts after the end of the administration of probiotics (119). On the other hand, the amount of *Bacteroides* in a different study was revealed as lower in patients with dementia, with a higher number of “other” bacteria strains (120).

In the aforementioned study concerning MCI patients, the efficacy and safety of *Lacticaseibacillus plantarum* C29-fermented soybean as a nutritional supplement for cognitive enhancement was assessed (114). The assessed total composite score included working memory, verbal memory and attention (114). The improvement in the combined cognitive functions at the end of the study was greater in the test group in comparison to the placebo group (114). There were also some adverse events observed in both groups: stomach aches and erectile dysfunction (114). In the placebo group irregular bowel movement was also observed (114). Dizziness, headaches, gastritis and seborrheic dermatitis occurred in the test group (114).

After administration of *Lacticaseibacillus helveticus* in older adults experiencing mild memory deficits, an improvement in the Repeatable Battery for Assessment of Neuropsychological Status (RBANS) test score was observed (121). Attention, coding and delayed memory were also found to improve after this intervention (121).

### Healthy Individuals

Eating habits and specific diets may contribute to an improvement in cognitive functions by positively affecting the composition of intestinal microbiota (122). A preliminary study on neurologically healthy older adults revealed an association between benefits for cognitive function and higher proportions of *Firmicutes* and *Verrucomicrobia* in the intestine (123). On the other hand, elevated amounts of *Bacteroidetes* and *Proteobacteria* might be affecting cognitive performance negatively (123).

*Lacticaseibacillus* contributes to intestinal health and is one of the most commonly administered groups of probiotics (124). In healthy women, a 3-month-long administration of *Lacticaseibacillus plantarum* P8 led to an improvement in the speed of social emotional cognition (124). However, in men, there was no significant difference between the test and placebo groups after the intervention (124).

A similar study was performed on healthy people, who were divided into three groups: ‘probiotic,’ ‘placebo,’ and ‘no intervention’ (125). This time probiotic supplementation lasted for 4 weeks and, beside *Lacticaseibacillus*, also *Bifidobacterium* was administered (125). The probiotic recipients experienced a growing positive affect and weakening susceptibility to depression, which was revealed in depression and anxiety questionnaires (125). They were also less likely to change their decisions after an occurrence of negative stimuli, and they improved their accuracy in response to unpleasant stimuli in contrast to ‘placebo’ and ‘no intervention’ group (125).

In another study, *Lacticaseibacillus* was used as a parabiotic (121). Paraprobiotics, just like probiotics, also benefit the host’s health. They consist of non-viable microbial cells, cell fractions or crude cell extracts (121). The latter makes their form more stable than that of probiotics (121). A 12-week-long supplementation of a low-dose *Lacticaseibacillus helveticus* in healthy elderly patients slightly improved their information-processing accuracy during the intervention (121). However, changes to all other cognitive functions – such as memory, interference, and vigilance – were not significant (121).

One of the components of *Lacticaseibacillus helveticus*-fermented milk is lactononadecapeptide (126). This substance was hypothesized to lead to an improvement in attention score, delayed memory score, coding score and in the RBANS total score among healthy, middle-aged adults after an 8-week long intake (126). However, the results in some domains – such as figure recall scores – in the placebo group were also elevated at the end of the study (126).

Research conducted in Wales showed that healthy volunteers experiencing a low mood at the beginning of the study who consumed *Lacticaseibacillus casei*-containing drink for 20 days, noted a mood improvement in the questionnaire based on Profile of Mood States (POMS) in comparison to a placebo group (127). Surprisingly though, patients who consumed probiotics had worse memory scores than the group who drank placebo (127). There was no statistical difference in verbal fluency between both groups (127).

When it comes to research on other types of bacteria then *Lactobacilli*, a study on 22 healthy male participants supplemented with *Bifidobacterium longum* 1714 strain was performed (128). Before and after the intervention, errors on the paired associate learning test were measured (128). Both the supplemented, and the placebo group, achieved better results after the intervention (128). This tendency was especially evident in the supplemented group (128).

### Hopes and Limitations of Microbiota Targeted Interventions

It is worth remembering that probiotics are unable to permanently colonize the gut after their discontinuation and their effect may finish in a few days to 2 weeks (129). On the contrary, the effect of prolonged consumption of probiotics by humans remains unknown (78). The majority of studies showed microbiota-targeted interventions to be safe to use, although, in one review, some adverse effects of probiotic supplementation have been described (130). Due to the fact that carbohydrates ferment in contact with probiotics, a bacterial overgrowth in the gut is induced, resulting in the D-lactic acid production, which leads to increased gas production in the gut (130). D-lactic acid production is also responsible for brain fogginess and neurocognitive symptoms or chronic fatigue syndrome (130).

Besides the time of action and side effects, individual efficacy also should be considered. According to research, the efficacy of probiotics across human populations varies depending on genetics, ethnicity, age, health status, cultural traditions and geographic locations (131). Most of these factors are associated with a host diet which is likely to have significant effects on their survival (131). Moreover, gut microbiota of humans are highly individual, and compositional differences might define who is likely to respond to probiotics therapy and who not (132).

When it comes to a host’s diet, it is worth considering not only dietary supplements, but also everyday food products, since some of them fulfill the definition of probiotics or prebiotics (129). Consumption of fermented food appears to alter the composition of gut microbiota (129). Commonly consumed fermented food has been also linked with lowering depressive (133) and anxiety (134) symptoms. Although, the efficacy of fermented food might be affected by variable levels of microbial cells, ranging from none to more than a billion colony-forming units per gram of a foodstuff (135). Moreover, the quality of microbes present in fermented food also raises doubts and may contribute to contradictory results regarding mental health (135); only psychiatric patients may benefit more from fermented food consumption (136). Those concerns explain to a certain extent why the recent meta-analysis does not support the use of fermented food intervention for cognitive functions (105).

The next challenge for the use of probiotics in cognitive impairment is establishing a safe and effective dose for humans. The actual dose of live microbes might be also affected by consumption of fermented food or food-derived prebiotics, which are hardly measurable on an everyday basis (135). Huang et al. (83) attempted at adjusting an adequate dose, based on the animal model, but further research is definitely needed in that regard (83).

To sum up, besides the difficulties mentioned above, probiotics should be further examined as a potential therapy for neuro-psychiatric diseases. Probiotics influenced the CNS in almost every analyzed study (89). However, a meta-analysis on the use of prebiotics, probiotics, fermented food interventions for cognitive performance revealed that the use of probiotics appears to have no significant effects on global cognition in humans (104). Studies included in the meta-analysis concerned mostly healthy participants, but there were also those suffering from various diseases, from neuropsychiatric ones to cirrhosis or fibromyalgia. 14 of 22 studies reported a significant improvement in specific cognitive domains (104).

This is too early so far to find a clear answer if microbiota targeted interventions are appropriate for alleviating cognitive impairment in depression because – according to our knowledge – there was only one study tackling this problem directly (99). This research revealed a positive response to the probiotic therapy (99). Unfortunately, many clinicians use screening and assessment tools that are not suited for measuring cognitive impairment in patients with depression (132). The new THINC-it assessment tool is the first instrument that provides objective and subjective data on dysfunctions in all the cognitive domains commonly affected by depression (132). However, our goal was to hypothesize about probiotics therapy in cognitive impairment among depressive patients and to encourage clinicians to perform more well-designed research on the matter.

In the case when THINC-it is unavailable, there are other tests assessing cognitive performance (105). For instance, MMSE, in which patients who achieve a score of at least 24 out of 30 are classified as those without cognitive impairment (137). There are also other tests used in assessment of cognitive functions, such as CogState Brief Battery, Block Design Test or Digit Symbol Test (105). Using the validated tests seems to be the most viable. Since researchers have used a wide variety of tests for various cognitive domains so far, performing a meta-analysis to assess the efficacy of microbiota targeted interventions is challenging (105).

Moreover, making conclusions about the effectiveness of probiotics in the treatment of cognitive impairment is also demanding with regard of probiotic strains. In the study performed by Rudzki et al. (99), which is particularly relevant to this review, as it considers depression, there was only one strain used – *Lactobacillus plantarum* 299v (99). According to the meta-analysis of the anxiolytic effect of probiotics (138) only *Lactobacillus rhamnosus* was found to significantly reduce anxiety-like behavior. In addition, multi-strain, but well-defined probiotics appear more effective (139).

Despite the difficulties, it is not surprising that probiotics may be helpful in impared cognition in the course of depression, as they are capable of alleviating multiple disorders (140). Gut microbiota-based interventions work according to a multi-target theory attributed largely to dietary interventions (141). As such probiotics may restore the overall healthy microbiome which appears crucial to achieve the optimal homeostasis of the human considered as a holobiont (126), recovering the health of the entire planet (142).

## Conclusion

Depression, due to its growing occurrence, is a great challenge for contemporary medicine. Most depressive patients also suffer from cognitive impairment related to their primary disease. Moreover, patients often do not achieve their previous cognitive functions despite their recovery from depression. It constitutes grave obstacles to a successful return to work and social life, which might in turn result in a consequential decrease in mood. Therefore, this public health problem should be particularly highlighted.

The mechanisms of cognitive impairment during depressive episodes are widely hypothesized. These symptoms might be explained by excessive oxidative stress and inflammation, which corresponds with a growing popularity of a theory claiming depression to be an inflammatory disease. Inflammatory processes seem to drive a decrease in neuroplasticity and a damage of brain structures, mainly the hippocampus. Other hypotheses with similar consequences are associated with hypercortisolemia, deposition of beta-amyloid plaques in the CNS or brain ischemia. The latter two may be of particular significance in the elderly.

Since probiotics are thought to have a procognitive mechanism of action, they are hypothesized to be a plausible treatment option for cognitive impairment among depressive patients. A single randomized controlled trial exists to support the effect of probiotics in alleviation of cognitive symptoms in depressed patients. This approach seems to be promising, since other research on humans and animals showed probiotics to be antioxidants and anti-inflammatory agents, based on their mechanism of action. Additionally, research on animals revealed mechanisms in which probiotics normalize the HPA axis. Studies on animals also showed a decline in beta-amyloid plaques, but despite neuro-inflammation, the causal pathways, which explain this phenomenon, remain unknown.

However, it is worth mentioning that most of the research was performed on animals, which served as models for human-specific conditions or even underwent invasive procedures. Attempts to equate human and animal cognitive abilities or mood stages also raise doubts. Nevertheless, most studies on animal models evidenced an improvement in cognition with little to no side effects. That – among others – raises an urgent need for well-planned clinical trials, especially on representative groups of patients.

Until now, the value of probiotics in the treatment of cognitive impairment in depression remains inconclusive. However, their complex mechanism of action reminds us of the great strength and potential of natural solutions in medicine.

## Author Contributions

MD and NB contributed to the literature collection, literature review, and manuscript preparation. KZ contributed to the linguistic editorial and manuscript preparation. EK contributed to the research supervision. MK contributed to the study design, literature collection, literature review, manuscript preparation, and research supervision. All authors contributed to the article and approved the submitted version.

## Conflict of Interest

The authors declare that the research was conducted in the absence of any commercial or financial relationships that could be construed as a potential conflict of interest.

## Publisher’s Note

All claims expressed in this article are solely those of the authors and do not necessarily represent those of their affiliated organizations, or those of the publisher, the editors and the reviewers. Any product that may be evaluated in this article, or claim that may be made by its manufacturer, is not guaranteed or endorsed by the publisher.
